# Total Ophthalmoplegia as an Early Manifestation of Herpes Zoster Ophthalmicus in an Elderly Patient With Asthma: A Case Report

**DOI:** 10.7759/cureus.113115

**Published:** 2026-07-21

**Authors:** Ela Gazal, Merve Karabulut, Ümit Türsen

**Affiliations:** 1 Dermatology, Mersin University Hospital, Mersin, TUR

**Keywords:** asthma, herpes zoster ophthalmicus, inhaled corticosteroid, ocular motor cranial neuropathy, periorbital zoster, th1/th2 imbalance, total ophthalmoplegia

## Abstract

Herpes zoster ophthalmicus (HZO) may cause ocular surface disease, but ocular motor cranial neuropathy and total ophthalmoplegia are rare. These neurological deficits generally develop after the cutaneous eruption, making their presence at initial presentation unusual. Asthma has recently gained attention as a risk factor for herpes zoster, potentially through Th2-skewed inflammation, relative Th1-mediated antiviral immune insufficiency, and impaired cellular control of latent varicella-zoster virus (VZV).

We report a 71-year-old man with poorly controlled asthma who presented with a one-day history of a right-sided vesicular eruption following four days of severe burning pain in the right scalp and periorbital region. Total ophthalmoplegia was already present at initial evaluation. He had no known diabetes mellitus, hypertension, malignancy, systemic immunosuppression, recent infection, biologic therapy, or chronic systemic corticosteroid use. His only notable comorbidity was asthma, for which fluticasone furoate/umeclidinium/vilanterol had been prescribed, although he used it irregularly. The right eye was fixed, with total ptosis, absent spontaneous movement, and marked restriction in all gaze directions. Presenting uncorrected visual acuity was 0.3 in the right eye and 0.8 in the left eye using decimal notation, and anisocoria was present with absent direct and consensual light reflexes. The diagnosis of HZO was clinical; VZV polymerase chain reaction testing was not performed because of the characteristic painful unilateral vesicular eruption in the ophthalmic dermatome. Contrast-enhanced orbital magnetic resonance imaging was normal, and laboratory evaluation did not reveal any other systemic risk factor, including a negative HIV and viral hepatitis panel. The patient received intravenous acyclovir at 10 mg/kg every eight hours for 21 days, with daily renal function monitoring, as well as a short course of methylprednisolone and topical dermatologic and ophthalmologic therapies. The cutaneous lesions regressed, and the corneal epithelial defect closed; however, complete ptosis and ophthalmoplegia persisted, and visual acuity remained the same as the baseline in both eyes at the fourth-week follow-up.

This case demonstrates that total ophthalmoplegia may be present at the initial presentation of HZO. Advanced age was the patient’s major established risk factor, while asthma was his only chronic comorbidity. Asthma may represent a contributing clinical context, but a causal relationship with complicated HZO cannot be inferred from a single case.

## Introduction

Herpes zoster ophthalmicus (HZO) results from reactivation of latent varicella-zoster virus (VZV) involving the ophthalmic division of the trigeminal nerve. Ocular involvement may include conjunctivitis, keratitis, uveitis, retinitis, optic neuropathy, and neuro-ophthalmic complications; however, ocular motor cranial neuropathy remains uncommon [1,2].

Ophthalmoplegia related to HZO has been described in several clinical patterns, including isolated ocular motor nerve palsies, combined cranial nerve involvement, orbital apex syndrome, orbital myositis, and total ophthalmoplegia [2-10]. Most reports emphasize delayed onset after the cutaneous eruption, although simultaneous or early presentations have also been documented [3,4,6,7].

Asthma is not traditionally considered overt immunosuppression, but epidemiologic and mechanistic studies support an association between asthma and increased herpes zoster risk [11-15]. Th2-predominant inflammation, relatively reduced Th1 antiviral responses, impaired innate antiviral signaling, and corticosteroid exposure have been proposed as contributing factors [13-16]. We report an elderly patient with HZO complicated by total ophthalmoplegia at admission, in whom asthma was the only notable comorbidity.

## Case presentation

A 71-year-old man presented to our dermatology clinic with a one-day history of vesicular eruption following four days of severe burning pain in the right scalp and periorbital region. The eruption began on the right parietal scalp and spread within the same day to the right frontoparietal and periorbital region. Before dermatologic evaluation, he had visited neurology and dentistry because of the severity of pain, but no definitive diagnosis had been established.

His medical history was notable for poorly controlled asthma. He had been prescribed inhaled fluticasone furoate/umeclidinium/vilanterol but reported irregular use. He had no known diabetes mellitus, hypertension, malignancy, systemic immunosuppression, recent infection, biologic therapy, transplantation history, chemotherapy, or chronic systemic corticosteroid use. He had not received a herpes zoster vaccination, while his childhood varicella vaccination status was unknown.

Dermatologic examination showed newly developed grouped vesicles on an erythematous background involving the right parietal scalp, forehead, and periorbital region, consistent with HZO in the right ophthalmic dermatome. The Hutchinson sign was absent. The diagnosis was based on the characteristic painful unilateral vesicular eruption in the ophthalmic distribution; VZV polymerase chain reaction testing was not performed.

**Figure 1:**
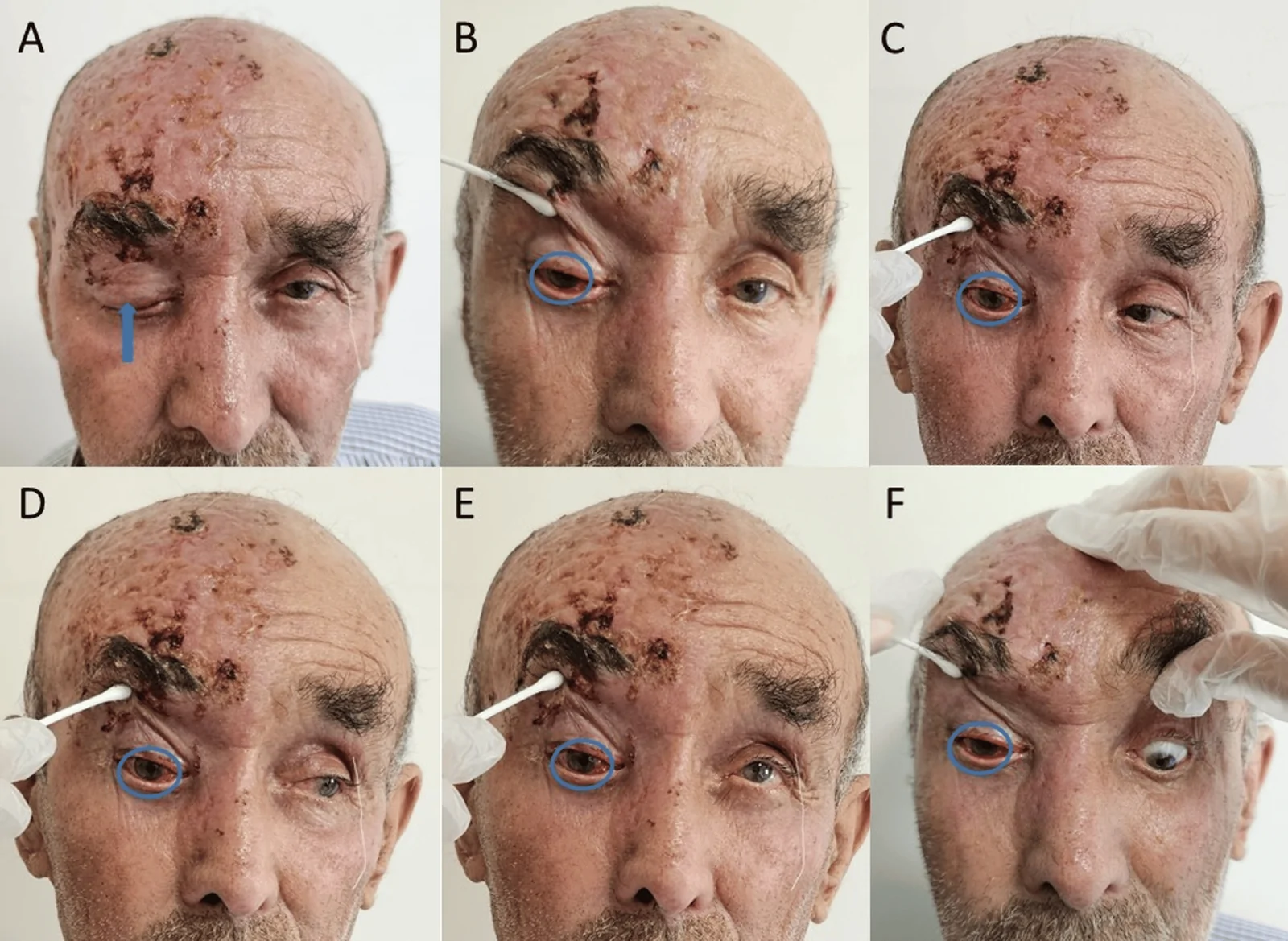
Herpes zoster ophthalmicus with persistent total ophthalmoplegia on day 14 of intravenous acyclovir treatment, 19 days after the onset of pain and burning symptoms. (A) Residual right ophthalmic-dermatome involvement with resolving erythema, partially healed erosions, and adherent yellow-brown and hemorrhagic crusts over the right frontoparietal scalp, forehead, upper eyelid, and periorbital region. The arrow indicates complete right-sided ptosis. Sparing of the nasal tip indicates the absence of the Hutchinson sign. (B) Primary gaze examination following manual retraction of the right upper eyelid demonstrates the affected eye fixed in the primary position. (C-F) Attempted right gaze, left gaze, upgaze, and downgaze, respectively. The left eye moves appropriately in the requested directions, whereas the right eye shows absent abduction (C), adduction (D), elevation (E), and depression (F). Identical blue circles identify the affected right eye and highlight its unchanged position across the attempted gaze directions, demonstrating total ophthalmoplegia.

**Figure 2:**
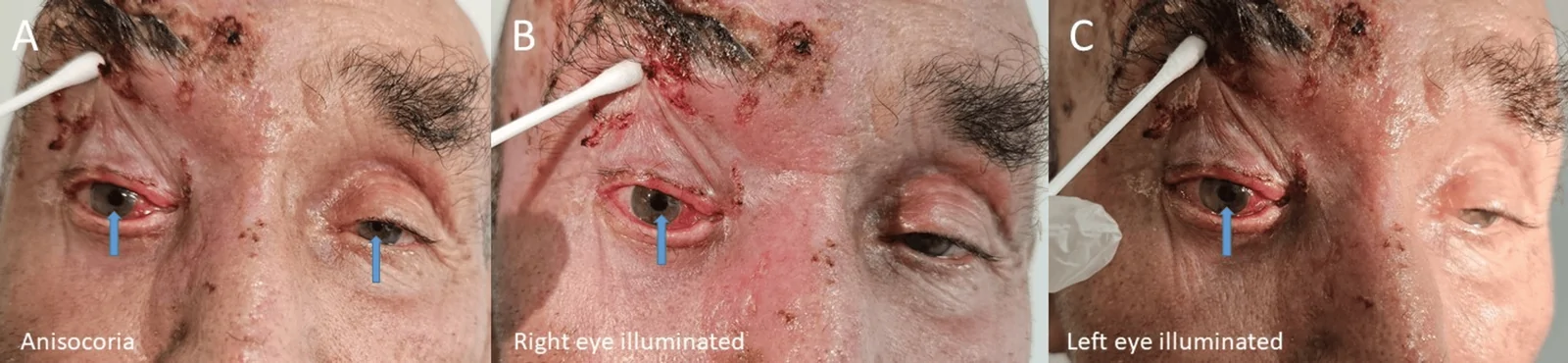
Pupillary abnormalities associated with right-sided total ophthalmoplegia on day 14 of intravenous acyclovir treatment. The right upper eyelid was manually retracted throughout the examination. (A) Anisocoria under ambient illumination, with the right pupil larger than the left; blue arrows identify both pupils for comparison. (B) During illumination of the right eye, the right pupil fails to constrict, demonstrating an absent direct pupillary light reflex. (C) During illumination of the left eye, the right pupil fails to constrict consensually, demonstrating an absent right consensual pupillary light reflex. Blue arrows in panels B and C identify the non-reactive right pupil.

Because of complete ptosis, the right upper eyelid was manually retracted for examination. Presenting uncorrected visual acuity was 0.3 in the right eye and 0.8 in the left eye using decimal notation; best-corrected visual acuity was not available. The right eye was fixed in the primary position and showed severe restriction of movement in all gaze directions. Clinical photographs obtained during cardinal gaze examination demonstrated these ocular motor findings (Figure 1). Anisocoria was present. The right pupil was non-reactive to both direct and consensual light stimulation, whereas direct and consensual responses of the left pupil were preserved. The corresponding pupillary findings are shown in Figure 2. Exact pupil diameters were not recorded, and a formal swinging-flashlight test for a relative afferent pupillary defect was not documented.

Ophthalmologic evaluation also revealed conjunctival hyperemia and chemosis, corneal edema, punctate epithelial keratitis, mucus plaques, and a small paracentral temporal epithelial defect in the right eye. Fundus examination showed no evidence of acute retinal necrosis in the visible retinal areas. Intraocular pressure was documented qualitatively as elevated in the right eye and normal in the left eye; numerical measurements were not available. The remainder of the left-eye examination was unremarkable.

Contrast-enhanced orbital and maxillofacial MRI examinations were normal; diffusion-weighted imaging likewise showed no abnormality. The globes, orbital osseous structures, extraocular muscles, optic nerves, optic tracts, optic chiasm, and intraconal and extraconal compartments were normal, with no pathological orbital signal abnormality. Laboratory investigations were within normal limits and did not reveal an additional systemic risk factor, including negative serological testing for human immunodeficiency virus, hepatitis B virus, and hepatitis C virus.

Because of the severe neuro-ophthalmic presentation with total ophthalmoplegia and concurrent ocular involvement, intravenous acyclovir was administered at 10 mg/kg every eight hours (700 mg every eight hours based on a body weight of 70 kg) for 21 days. Renal function was normal at baseline and remained stable during daily monitoring throughout treatment; therefore, no dose adjustment was required. The patient also received methylprednisolone 40 mg/day for the first three days. Dermatologic treatment included topical fusidic acid, topical rifampicin-based therapy, and wet dressings. Ophthalmologic treatment included topical ganciclovir, topical moxifloxacin, frequent lubricants, and topical anti-inflammatory therapy.

Following treatment, the cutaneous lesions crusted and regressed, and the corneal epithelial defect resolved. However, at the latest ophthalmologic evaluation at week 4, uncorrected visual acuity remained at baseline in both eyes. Complete ptosis and severe restriction of right extraocular movements in all directions persisted without documented improvement. No further long-term follow-up was available.

## Discussion

HZO-associated ophthalmoplegia is uncommon but clinically important because it may indicate ocular motor cranial neuropathy, orbital inflammation, orbital apex involvement, cavernous sinus inflammation, or overlapping inflammatory mechanisms [1-10]. In a classic review, Marsh et al. described external ocular motor palsies in ophthalmic zoster, and subsequent reports have expanded the spectrum to include total ophthalmoplegia, orbital myositis, and orbital apex syndrome [2-10]. In a dermatology-based series of 259 HZO inpatients, oculomotor nerve palsy was identified in five patients, all elderly and comorbid but not immunosuppressed [10].

The timing in our patient is notable. In the review by Sanjay et al., HZO preceded ophthalmoplegia in most patients, with a mean interval of 9.5 days [3]. Im et al. reported total ophthalmoplegia seven days after onset of ophthalmic zoster, while Shin et al. described total ophthalmoplegia following HZO in an elderly patient [6,7]. DeLengocky and Bui reported total ophthalmoplegia with pupillary involvement as an initial presentation of HZO, showing that early neuro-ophthalmic involvement is possible [8]. In our patient, total ophthalmoplegia was already present when he reached dermatology, although the vesicular eruption had been present for only one day.

Several mechanisms have been proposed for HZO-associated ophthalmoplegia. These include direct viral cytopathic injury, immune-mediated neuritis, VZV-related vasculitis, microinfarction of ocular motor nerves, orbital myositis, and inflammatory spread to the superior orbital fissure, cavernous sinus, or orbital apex [2-5,9,10]. Daswani et al. reported HZO with orbital myositis, oculomotor nerve palsy, and anterior uveitis, with MRI showing extraocular muscle and oculomotor nerve thickening [9]. Lee et al. described orbital apex syndrome secondary to HZO, with total ophthalmoplegia and optic nerve sheath enhancement [5]. In our patient, contrast-enhanced orbital MRI did not demonstrate extraocular muscle, optic nerve, or intraorbital abnormalities. Nevertheless, normal MRI does not exclude HZO-associated inflammatory ocular motor cranial neuropathy or microvascular injury, as small-caliber neural inflammation and microscopic vascular changes may remain below the resolution of routine imaging. In the absence of a radiologically evident orbital or structural process, radiographically occult inflammatory cranial neuropathy or a microvascular mechanism was considered most plausible, although the precise mechanism could not be established.

No standardized antiviral duration has been established specifically for HZO-associated ophthalmoplegia. Given the early total ophthalmoplegia and concurrent ocular involvement, a 21-day course was selected at the upper end of the two- to three-week treatment durations described for severe neuro-ophthalmic HZO [1].

Advanced age is a major established risk factor for herpes zoster because VZV-specific cell-mediated immunity declines with immunosenescence [1,14,15]. Therefore, the patient’s age of 71 years likely represented his principal susceptibility factor. Apart from age, however, asthma was his only chronic comorbidity identified after evaluation; he had no diabetes mellitus, hypertension, malignancy, systemic immunosuppression, or previous immunosuppressive treatment. Bronchial asthma has also been reported among the comorbidities of some patients with HZO-associated oculomotor nerve palsy, although its relevance to this complication remains uncertain [10].

Epidemiological studies have associated asthma with a modestly increased risk of herpes zoster [11-14]. Proposed mechanisms include Th2-predominant inflammation and relatively impaired Th1-mediated antiviral immunity [14,15]. The patient had also been prescribed an inhaled corticosteroid-containing combination therapy, although he used it irregularly. Evidence regarding the independent contribution of inhaled corticosteroids is inconsistent [13,14,16]. Accordingly, while older age constitutes the major risk factor for reactivated HZO, asthma and intermittent inhaled corticosteroid use may have provided a contributing clinical context; however, this association does not establish a causal relationship for either HZO reactivation or increased disease severity in this patient.

The most unusual feature of this case was that total ophthalmoplegia was already present at the initial dermatologic evaluation, when the vesicular eruption had been present for only one day. This finding underscores the importance of examining ocular motility, ptosis, and pupillary responses at the first assessment of patients with periorbital zoster. Asthma was the patient’s only chronic comorbidity, but its possible contribution should be interpreted cautiously in the context of advanced age and the limitations of a single case.

## Conclusions

HZO may rarely present with total ophthalmoplegia at initial evaluation rather than as a delayed complication. Early assessment of ocular motility, ptosis, visual acuity, and pupillary responses is therefore important in patients with periorbital zoster. In this patient, advanced age was a major established risk factor, while asthma was the only chronic comorbidity identified. This case suggests that asthma may represent a possible contributing clinical context for complicated HZO, but it does not establish a causal association. Further studies are required to clarify this relationship.
